# Supplementary material for: Diverse inhibitory projections from the cerebellar interposed nucleus
Source: eLife. 2021 Sep 20;10:e66231. doi: 10.7554/eLife.66231 (PMC8483738; doi:10.7554/eLife.66231)
Supplement: Supplementary file 1. [file elife-66231-supp1.docx]

| Region | Int-Vgat | Int^IO^-Vgat | Int^RN^ | Int-Ntsr1 | Int^RN^-Ntsr1 |
| --- | --- | --- | --- | --- | --- |
| Motor |  |  |  |  |  |
| Red nucleus (RN) | **O**  **+** | **O**  **++** | **++++** | **++++** | **++++** |
| Ventral anterior-lateral complex of the thalamus (VAL) |  | **+** | **O**  **++++** | **O**  **++++** | **O**  **++++** |
| Midbrain reticular nucleus (MRN) | **O**  **+** | **O**  **+** | **O**  **+++** | **O**  **+++** | **++++** |
| Tegmental Reticular nucleus (TRN) | **O**  **++** | **O**  **++** | **+++** | **+++** | **++++** |
| Ventral medial nucleus of the thalamus (VM) | **O** |  | **+++** | **+++** | **+++** |
| Superior colliculus (SC) | **O** | **+** | **O**  **+++** | **+++** | **++** |
| Gigantocellular reticular nucleus (GRN) | **OO**  **+** | **OO**  **+** | **+++** | **O**  **+++** | **O**  **++** |
| Pontine reticular nucleus (PRN) | **O**  **++** | **O**  **++** | **O**  **++** | **++** | **++** |
| Medullary reticular nucleus, ventral (MDRNv) | **OO**  **+** | **OO**  **+** | **O**  **++** | **O**  **++** | **O**  **++** |
| Periaquaductal grey (PAG) | **OO**  **+** | **OO** | **O**  **++** | **O**  **++** | **O**  **++** |
| Anterior pretectal nucleus (APN) | **O** |  | **++** | **++** |  |
| Magenocellular reticular nucleus (MARN) | **O**  **+** | **O**  **+** | **++** | **++** | **++** |
| Pontine grey (PG) | **O**  **+++** | **OO**  **+++** | **+** | **++** | **+** |
| Lateral reticular nucleus (LRN) | **O**  **++** | **O** | **OO** | **O**  **+** | **+** |
| Intermediate reticular nucleus (IRN) | **OO** | **O** | **OOO**  **+** | **OO**  **+** | **OO** |
| Spinal cord (SpC) | **+** |  |  | **+** | **++** |
| Inferior olive (IO) | **OOO**  **++++** | **OOO**  **++++** | **O**  **+++** | **+** |  |
| Medial vestibular nucleus (MV) | **OO**  **+** | **O**  **+** | **O**  **+** | **O**  **+** | **O** |
| Lateral vestibular nucleus (LAV) | **OO**  **+** | **OO**  **+** | **OO**  **+** | **OO**  **+** | **OO**  **+** |
| Superior vestibular nucleus (SUV) | **OOO**  **++** | **OO**  **+** | **OO**  **+** | **OO**  **+** | **O** |
| Nucleus Y (Y) | **OO**  **+** | **O** | **O** | **OO**  **+** | **O** |
| Parvicellular reticular nucleus (PARN) | **OO** | **O** | **OOO**  **+** | **OOO**  **+** | **OOO** |
| Spinal vestibular nucleus (SPIV) | **OOO**  **++** | **OO**  **+** | **OO**  **+** | **OO**  **+** | **OO**  **+** |
| Nucleus prepositus (PRP) | **OO** |  | **O** | **+** |  |
| Medullary reticular nucleus, dorsal (MDRNd) | **OO**  **+** | **O** | **OOOO**  **+** | **OOO** | **OOO** |
| Supratrigeminal nucleus (SUT) |  |  | **O** | **O** | **O** |
| Nucleus X (X) | **OO**  **+** | **O**  **+** | **OO** | **O** | **O** |
| Motor nucleus of trigeminal (V) | **O**  **+** |  | **OO** | **OO** | **OO** |
| Paragigantocellular reticular nucleus (PGRN) | **OO**  **+** | **OO**  **+** |  |  |  |
| Hypoglossal nucleus (XII) | **OO**  **+** | **OO**  **+** |  |  |  |
| Oculomotor nucleus (III) | **OO** | **O** |  |  |  |
| Facial motor nucleus (VII) | **O**  **+** |  |  |  |  |
| Medial nucleus of the cerebellum (MN) | **+** | **+** |  |  |  |
|  |  |  |  |  |  |
| Mixed |  |  |  |  |  |
| Zona incerta (ZI) | **O**  **+** | **O** | **++** | **+++** | **++** |
|  |  |  |  |  |  |
| Sensory |  |  |  |  |  |
| Parabrachial nucleus (PB) | **OO**  **++** | **O**  **+** | **OO** | **OO** | **OO** |
| Principal sensory nucleus of the trigeminal (PSV) | **OO**  **+** | **O** | **OO** | **OO** | **O** |
| Cuneate nucleus (CU) | **OOO**  **+** | **OO**  **+** | **O**  **+** | **OO**  **+** | **O** |
| Spinal nucleus of the trigeminal, oral (SPVo) | **OO**  **+** | **O** | **OO** | **OO** | **OO** |
| Nucleus of the solitary tract (NTS) | **OO**  **+** | **O**  **+** |  | **O**  **+** |  |
| Spinal nucleus of the trigeminal, interpolar (SPVi) | **OO**  **+** | **OO**  **+** | **OO** | **O** | **O** |
| Ventral posteromedial nucleus of the thalamus (VPM) | **O** | **O**  **+** | **+++** | **++** | **+** |
| Ventral posterolateral nucleus of the thalamus (VPL) |  |  | **+** | **+** | **+** |
| External cuneate nucleus (ECU) | **OO**  **+** | **O**  **+** | **O**  **+** |  |  |
| Spinal nucleus of the trigeminal, caudal (SPVc) | **OO**  **+** | **O** |  |  |  |
|  |  |  |  |  |  |
| Modulatory/ Affective |  |  |  |  |  |
| Ventral tegmental area (VTA) | **+** |  | **+++** | **+++** | **++** |
| Parafascicular nucleus (PF) | **+** |  | **+++** | **+++** | **++** |
| Central medial nucleus of the thalamus (CM) / Paracentral nucleus (PCN) |  |  | **O**  **++** | **OO**  **++** | **O**  **++** |
| Mediodorsal nucleus of the thalamus (MD) |  |  | **++** | **++** | **+** |
| Posterior complex of the thalamus (PO) |  |  | **+** | **++** | **+** |
| Nucleus raphe magnus (RM) |  |  | **+** | **++** | **+** |
| Pedunculopontine nucleus (PPN) | **+** |  | **O**  **+** | **+** |  |
|  |  |  |  |  |  |
| Cerebellar Cortex |  |  |  |  |  |
| Lobule 2 (Lob2) | **O** |  |  |  |  |
| Lobule 3 (Lob 3) | **OO** |  | **O** | **OO** | **O** |
| Lobules 4/5 (Lob 4/5) | **OO**  **+** | **O** | **OOO** | **OOO** | **OO** |
| Lobule 6 (Lob 6) | **O**  **+** |  | **OO** | **OO** | **O** |
| Lobule 7 (Lob7) | **O** |  | **O** |  |  |
| Lobule 8 (Lob 8) | **OO**  **+** |  | **O** | **O** |  |
| Lobule 9 (Lob 9) | **OOO**  **++** | **O** | **O** |  |  |
| Lobule 10 (Lob 10) | **O**  **+** |  |  |  |  |
| Flocculus (Fl) | **OO** |  |  |  |  |
| Paraflocculus (PFl) | **OO** | **O** |  | **O** | **O** |
| Paramedian (PM) | **OO**  **+** |  | **OOO** | **OOO** | **O** |
| Copula (Cop) | **OO**  **+** | **O** | **OOO** | **OOO** | **OO** |
| Crus 1 (Cr1) | **O** |  | **O** | **OO** | **O** |
| Crus 2 (Cr2) | **OO**  **+** | **O** | **OO** | **OO** | **OO** |
| Simplex (Sim) | **O** |  | **OOO** | **OOO** | **OO** |

Supplemental File 1. Anterograde tracing summary. Average RPS for all specimens in Int-Vgat (n = 6; including Allen specimen), Int^IO^-Vgat (n = 5), Int^RN^ (n = 4), Int-Ntsr1 (n = 6, including Allen specimen), and Int^RN^-Ntsr1 (n = 5). RPS depicted as symbols (+ for contralateral RPS, O for ipsilateral RPS). One symbol = avg RPS < 1, two symbols = avg RPS ≥ 1 and <2, three symbols = avg RPS ≥2 and <3, four symbols = avg RPS ≥ 3.
