# Supplementary material for: Diverse inhibitory projections from the cerebellar interposed nucleus
Source: eLife. 2021 Sep 20;10:e66231. doi: 10.7554/eLife.66231 (PMC8483738; doi:10.7554/eLife.66231)
Supplement: Transparent reporting form [file elife-66231-transrepform1.docx]

***eLife’s* transparent reporting form**

We encourage authors to provide detailed information *within their submission* to facilitate the interpretation and replication of experiments. Authors can upload supporting documentation to indicate the use of appropriate reporting guidelines for health-related research (see [EQUATOR Network](http://www.equator-network.org/%20)), life science research (see the [BioSharing Information Resource](https://biosharing.org/)), or the [ARRIVE guidelines](http://www.plosbiology.org/article/info:doi/10.1371/journal.pbio.1000412) for reporting work involving animal research. Where applicable, authors should refer to any relevant reporting standards documents in this form.

If you have any questions, please consult our Journal Policies and/or contact us: [editorial@elifesciences.org](mailto:editorial@elifesciences.org).

**Sample-size estimation**

- You should state whether an appropriate sample size was computed when the study was being designed
- You should state the statistical method of sample size computation and any required assumptions
- If no explicit power analysis was used, you should describe how you decided what sample (replicate) size (number) to use

Please outline where this information can be found within the submission (e.g., sections or figure legends), or explain why this information doesn’t apply to your submission:

This manuscript describes a variety of observations from anatomical tract tracing experiments that were not designed from the onset to produce quantitative datasets. While many neuroanatomical studies report single cases, including the present manuscript, we include reporting the extent to which replicates reproduce in part or in full the example dataset (e.g. mean projection strengths). This practice acknowledges the high sensitivity of results to injection site which is rarely if ever precisely replicated across individuals. To better illustrate consistency we used semiquantiative methods to describe projection strengths, and average them across samples (animals). These practices are described in the Methods and source data are provided. While we report statistical tests based on semi-quantitative scoring to bolster qualitative observations of differences across genotypes, we did not do power analyses as the nature of these datasets is not fundamentally quantitative, nor do the key observations rest on any statistical arguments.

**Replicates**

- You should report how often each experiment was performed
- You should include a definition of biological versus technical replication
- The data obtained should be provided and sufficient information should be provided to indicate the number of independent biological and/or technical replicates
- If you encountered any outliers, you should describe how these were handled
- Criteria for exclusion/inclusion of data should be clearly stated
- High-throughput sequence data should be uploaded before submission, with a private link for reviewers provided (these are available from both GEO and ArrayExpress)

Please outline where this information can be found within the submission (e.g., sections or figure legends), or explain why this information doesn’t apply to your submission:

Our N’s throughout the manuscript reflect biological replicates (different animals). Technical replication in these experiments reflects the reliability of making the same observation in the same sample across observers, which in this case were the first and senior authors. No outliers are excluded. We report criteria for inclusion/exclusion in the Methods based on injection site, observation of the presence of labelling to analyse.

**Statistical reporting**

- Statistical analysis methods should be described and justified
- Raw data should be presented in figures whenever informative to do so (typically when N per group is less than 10)
- For each experiment, you should identify the statistical tests used, exact values of N, definitions of center, methods of multiple test correction, and dispersion and precision measures (e.g., mean, median, SD, SEM, confidence intervals; and, for the major substantive results, a measure of effect size (e.g., Pearson's r, Cohen's d)
- Report exact p-values wherever possible alongside the summary statistics and 95% confidence intervals. These should be reported for all key questions and not only when the p-value is less than 0.05.

Please outline where this information can be found within the submission (e.g., sections or figure legends), or explain why this information doesn’t apply to your submission:

In the Results, we report which statistical tests are used and exact p values whenever appropriate. Raw data are presented throughout the study in figures, supplements and source data files as well as within a data repository. Along with reporting the tests used, we report the means, standard errors of the mean, and confidence intervals and identify methods for correction for multiple tests.

(For large datasets, or papers with a very large number of statistical tests, you may upload a single table file with tests, Ns, etc., with reference to sections in the manuscript.)

**Group allocation**

- Indicate how samples were allocated into experimental groups (in the case of clinical studies, please specify allocation to treatment method); if randomization was used, please also state if restricted randomization was applied
- Indicate if masking was used during group allocation, data collection and/or data analysis

Please outline where this information can be found within the submission (e.g., sections or figure legends), or explain why this information doesn’t apply to your submission:

Not applicable. The datasets were not collected from randomizable pools; experiments were not designed to test effectiveness of drugs or treatments, nor are claims made to such.

**Additional data files (“source data”)**

- We encourage you to upload relevant additional data files, such as numerical data that are represented as a graph in a figure, or as a summary table
- Where provided, these should be in the most useful format, and they can be uploaded as “Source data” files linked to a main figure or table
- Include model definition files including the full list of parameters used
- Include code used for data analysis (e.g., R, MatLab)
- Avoid stating that data files are “available upon request”

Please indicate the figures or tables for which source data files have been provided:

Figure 2 source data is in “Supplementary File 1 – Source data 1”

Figure 4 source data is in “Supplementary File 2 – Source data 1”

Figure 1-Figure Supplement 3 source data is in “Supplementary File 2”

“Supplementary File 1” Figure 6, and 7 source data is in “Supplementary File 3” and “Supplementary File 4”

Figure 6 – Figure Supplement 1 source data is in “Figure6_7_sourcedata1”
